# Targeting of prostate-specific membrane antigen for radio-ligand therapy of triple-negative breast cancer

**DOI:** 10.1186/s13058-019-1205-1

**Published:** 2019-10-22

**Authors:** Agnieszka Morgenroth, Ebru Tinkir, Andreas T. J. Vogg, Ramya Ambur Sankaranarayanan, Fatima Baazaoui, Felix M. Mottaghy

**Affiliations:** 1 Department of Nuclear Medicine, University Hospital Aachen, RWTH Aachen University, Pauwelsstrasse 30, 52074 Aachen, Germany; 2 Department of Radiology and Nuclear Medicine, Maastricht University Medical Center X, Maastricht, The Netherlands

**Keywords:** Triple-negative breast cancer, Prostate-specific membrane antigen, Radio-ligand therapy

## Abstract

**Background:**

Triple-negative breast cancer has extremely high risk of relapse due to the lack of targeted therapies, intra- and inter-tumoral heterogeneity, and the inherent and acquired resistance to therapies. In this study, we evaluate the potential of prostate-specific membrane antigen (PSMA) as target for radio-ligand therapy (RLT).

**Methods:**

Tube formation was investigated after incubation of endothelial HUVEC cells in tumor-conditioned media and monitored after staining using microscopy. A binding study with ^68^Ga-labeled PSMA-addressing ligand was used to indicate targeting potential of PSMA on tumor-conditioned HUVEC cells. For mimicking of the therapeutic application, tube formation potential and vitality of tumor-conditioned HUVEC cells were assessed following an incubation with radiolabeled PSMA-addressing ligand [^177^Lu]-PSMA-617. For in vivo experiments, NUDE mice were xenografted with triple-negative breast cancer cells MDA-MB231 or estrogen receptor expressing breast cancer cells MCF-7. Biodistribution and binding behavior of [^68^Ga]-PSMA-11 was investigated in both tumor models at 30 min post injection using μPET. PSMA- and CD31-specific staining was conducted to visualize PSMA expression and neovascularization in tumor tissue ex vivo.

**Results:**

The triple-negative breast cancer cells MDA-MB231 showed a high pro-angiogenetic potential on tube formation of endothelial HUVEC cells. The induced endothelial expression of PSMA was efficiently addressed by radiolabeled PSMA-specific ligands. ^177^Lu-labeled PSMA-617 strongly impaired the vitality and angiogenic potential of HUVEC cells. In vivo, as visualized by μPET, radiolabeled PSMA-ligand accumulated specifically in the triple-negative breast cancer xenograft MDA-MB231 (T/B ratio of 43.3 ± 0.9), while no [^68^Ga]-PSMA-11 was detected in the estrogen-sensitive MCF-7 xenograft (T/B ratio of 1.1 ± 0.1). An ex vivo immunofluorescence analysis confirmed the localization of PSMA on MDA-MB231 xenograft-associated endothelial cells and also on TNBC cells.

**Conclusions:**

Here we demonstrate PSMA as promising target for two-compartment endogenous radio-ligand therapy of triple-negative breast cancer.

## Background

Development of prostate-specific membrane antigen (PSMA) addressing small molecules initiated application of their radiolabeled derivatives for theranostics of prostate cancer (PrCa) [1]. Gene expression analysis revealed PSMA presence in several cancer types leading to increasing acceptance of PSMA as a target for positron emission tomography/computer tomography (PET/CT) imaging in patients [2]. Due to the enzymatic activity and its role during neo-angiogenic processes, PSMA becomes an attractive target for numerous solid tumor entities including glioma, thyroid, bronchial, hepatocellular, ovarian, and breast cancer. Unlike in prostate cancer, PSMA expression is preferentially presented in endothelial cells of tumor-associated neo-vasculature, with no endothelial expression under physiological conditions [3–6]. Thus, addressing PSMA presents now one of the most specific and effective therapeutic approaches, especially for tumor entities lacking targetable cell surface markers. Among them, the triple-negative breast cancer (TNBC) is standing out because of its extreme aggressive progression and the absence of druggable receptors like the estrogen and progesterone receptors, and of the human epidermal growth factor receptor 2 (Her2). Moreover, TNBC occur frequently in younger women, many of them carrying BRCA-1 mutations. Due to the phenotype, the possibilities for treatment are limited, as endocrine therapy with tamoxifen or aromatase-inhibitors and anti-Her2 therapy with trastuzumab are ineffective. The low 5-year survival rate of 77% vs. 93% for non-TNBC visualizes the urgent need for the development of more efficient therapy options [7]. Many therapeutic concepts using, e.g., poly (ADP-ribose) polymerase (PARP) inhibitors (iniparib, olaparib) or VEGF inhibitors (bevacizumab), have been developed for an improved treatment of the TNBC [8–10]. Even though these strategies did not yield an expected increase in cancer cell responsiveness, they proof the potential of targeting neo-angiogenesis and the potential activity of DNA damaging pharmaceuticals.

Here we evaluate the potential of PSMA as vascular target for radio-ligand therapy of triple-negative breast cancer. We investigate PSMA expression directly on the breast cancer cells and its impact on angiogenetic processes. In in vivo studies, we evaluate the efficacy of radiolabeled PSMA-ligand for addressing of TNBC xenograft.

## Materials and methods

### Chemicals

Chemicals and solvents were purchased from Sigma-Aldrich (St. Louis, MO, USA) and Merck (Germany) or otherwise as indicated. Used water and acetonitrile for reagents were from Merck. For staining, PSMA-specific antibody (Abcam Cat# ab19071, RRID:AB_444751) and the goat anti mouse DyLight488 antibody (Abcam Cat# ab96871, RRID:AB_10680543) were used. The CD31-specific antibody (Cat# MA3100, RRID:AB_223516) was purchased from ThermoFisher Scientifics. Precursors PSMA-11 and PSMA-617 were purchased from ABX (Germany) and Endocyte (USA). The nuclides ^68^Ga and ^177^Lu were supplied by iThemba (South Africa, generator system) and IDB (The Netherlands), respectively.

### Radiochemistry

Both radiopharmaceuticals, [^68^Ga]-PSMA-11 and [^177^Lu]-PSMA-617, were produced by a routine procedure primarily used for patient care. For this purpose, a cassette synthesizer from Scintomics type GRP 3 V was used with cassettes from ABX dedicated to the two radionuclides (SC-01 for ^68^Ga using HEPES buffer and SC-05 for ^177^Lu using acetate buffer during labeling). Briefly, 10 mL of ^68^GaCl_3_ containing generator eluate (0.6 M) was diluted with water and trapped on a cation exchange SPE cartridge, eluted with 5 M NaCl, and added to the reactor containing precursor PSMA-11 and HEPES. After labeling reaction (120 °C, 10 min), HEPES was removed using reversed phase SPE extraction. The product was eluted by aqueous EtOH followed by formulation with PBS. Purchased ^177^LuCl_3_ (< 1 mL) was transferred to the reactor containing precursor PSMA-617 and acetate buffer. After 20-min reaction at 100 °C, the solution was quenched by a saline solution containing DTPA. Radiochemical purities were > 95%, respectively.

### Cell lines and culture conditions

The following human breast cancer cell lines were used: MDA-MB 231 (ATCC Cat# CRM-HTB-26, RRID:CVCL_0062; Her2^−^, PR^−^, ER^−^) and MCF-7 (ATCC Cat# CRL-12584, RRID:CVCL_0031, Her2^−^, PR^−^, ER^+^). The cancer cell lines were routinely maintained in Eagle minimum essential medium (Sigma-Aldrich) supplemented with 10% FBS and 1% penicillin–streptomycin and 2 mmol/L l-glutamine. The endothelial cells HUVEC (ATCC Cat# CRL-1730, RRID:CVCL_2959) were cultured in F-12 K Medium supplemented with 2% FBS, 10 mmol/L l-glutamine, 5 ng/mL recombinant human (rh) basic fibroblast growth factor, 5 ng/mL rh epithelial growth factor, 5 ng/mL rh VEGF, 15 ng/mL rh insulin-like growth factor-1, 50 mg/mL ascorbic acid, 1 mg/mL hydrocortisone hemisuccinate, and 0.75 U/mL heparin sulphate (all reagents from Lifeline Cell Technology). All cell lines were authenticated by ATCC using the short tandem repeat-based DNA fingerprinting and multiplex PCR. The cell lines were tested be-weekly for mycoplasma contamination using a PCR analysis (PromoCell). All cell lines were fed twice a week and split with trypsinization when the cells were confluent. All cell cultures were kept at 37 °C in a humidified environment of 5% CO_2_ atmosphere. All cell experiments were executed within 6 weeks after thawing. For induction of hypoxia, the cells were treated with 150 μM cobalt chloride (CoCl_2_) for 24 h.

### Cell uptake experiments with breast cancer cells

For cell uptake experiments, breast cancer cells were seeded with 1 × 10^5^ cell per well in a 6-well plate at 24 h prior to incubation with [^68^Ga]-PSMA-11 (0.5 MBq/well). For the hypoxic condition, the cells were treated with CoCl_2_ (100 μM for 48 h) prior to incubation with [^68^Ga]-PSMA-11 (0.5 MBq/well). The co-incubation with the irreversible PSMA inhibitor PMPA (10 μM) was performed as a proof of the tracer binding specificity. After 1 h and 4 h, the cells were washed twice with PBS and trypsinized. The cell-associated radioactivity was measured in a gamma counter (Wizard2, PerkinElmer, USA). The experiments were carried out twice in triplicates (*n* = 6).

### Generation of breast cancer-conditioned media

For investigation of breast cancer cells and their impact on endothelial tube formation, HUVEC cells were incubated in breast cancer cell-conditioned media. These were harvested from MDA-MB231 and MCF-7 cells 24 h after the cells achieved 80% confluence. The cell media were centrifuged for 10 min at 3000×*g* and strained through a 0.20-μm filter.

### Cell uptake experiments with endothelial cells

The binding experiments with HUVEC cells were performed directly after tube formation assay. For this, wells were covered with cold Matrigel. After 10-min incubation at room temperature and 30-min incubation at 37 °C, HUVEC cells were seeded with 1 × 10^5^ cells per well in 300 μl HUVEC growth medium (EBM-2, supplemented with growth factors), MDA-MB231-conditioned medium, MCF-7-conditioned medium, or plain growth medium of both breast cancer cell lines (RPMI, negative control). The cells were incubated at 37 °C and 5% CO_2_ for 4 h, a time frame required for tube formation. Subsequently, the cells were incubated with [^68^Ga]-PSMA-11 (0.5 MBq/well) for 1 h at 37 °C and 5% CO_2_. The cell-associated radioactivity was measured in a gamma counter. The experiments were carried out twice in triplicates (*n* = 6).

### PSMA and CD31 staining of endothelial tubes

The HUVEC tubes were fixed for 15 min in 2%PFA/1% glutaraldehyde. After washing with PBS, HUVEC cells were permeabilized with 0.1% Triton-X100 in PBS for 5 min. After repeated washing with TBS, the cells were blocked with 3%BSA/PBS for 30 min at room temperature. For staining, the tubes were incubated with CD31-specific antibody (1:100) or with PSMA-specific antibody (1:250) overnight at 4 °C. After washing with PBS (3 times), the tubes were incubated with Dy-Light 488-labeled antibody (1250) for 1 h at room temperature. Finally, the tubes were washed with PBS (3 times) and coated with DAPI containing Vectashield. The staining was visualized by fluorescence microscopy (Axio Scope A, Zeiss).

### Flow cytometric analysis of PSMA expression

The expression of PSMA was investigated using flow cytometry (FACS, Cytomics FC 500, Beckman Coulter) by staining with PSMA-specific antibody, corresponding Ig control antibody, and fluorochrome-labeled secondary antibody. The cells were fixed in acetone/methanol for 5 min at − 20 °C. The cells were incubated with PSMA-specific antibody for 1 h at room temperature. After washing steps, the samples were incubated with Dy-Light488-labeled secondary antibody for 1 h at room temperature. After washing, the cells were measured on cytometer, and the data were analyzed using CXP Software (Beckman Coulter).

### Evaluation of anti-angiogenic potential of [^177^Lu]-PSMA-617

To evaluate the anti-angiogenic potential of PSMA targeting approach, the endothelial HUVEC cells were incubated with ^177^Lu-labeled PSMA-L. For this, HUVEC cells were seeded in a 12-well plate. One day later, the cells were incubated for 5 days in EBM-2-, RPMI-, MDA-MB231-, or MCF-7-conditioned media supplemented with [^177^Lu]-PSMA-617 (1 MBq/well). The viable cells were counted and seeded for the tube formation in the Matrigel-coated cover slips. After 4-h incubation at 37 °C and 5% CO_2_, the tubes were fixed and visualized by bright field microscopy (Axio Scope A, Zeiss).

### Animal study

Female NMRI-Foxn1nu/Fox1nu mice (MGI Cat# 2662818, RRID:MGI:2662818) were injected subcutaneously into the flank with MDA-MB231 (2 × 10^6^) cells or MCF-7 cells (2 × 10^6^). For μPET analysis, the animals received intravenously 10 MBq [^68^Ga]-PSMA-L. The molecular imaging of [^68^Ga]-Ga-PSMA-11 biodistribution was performed at 30 min post injection by using the μPET (Triumph II PET/SPECT/CT, Trifoil, USA). The PET images were reconstructed using the iterative OSEM3D/MAP (OSEM3D 2 iterations, MAP 18 iterations) algorithm. The region of interests (ROI) was manually drawn around tumor and within muscle tissue using PMOD software (RRID:SCR_016547). For quantification of tracer accumulation in the lesions, the target_mean_-to-background_mean_ (T/B) ratios were calculated. All animal experiments were done in accordance with national and local regulations for animal and radiation protection and good experimental practice (approved by Ethics committee LANUV in Recklinghausen,).

### Staining of tissue sections

Formalin-fixed, paraffin-embedded tissue sections (2 μm thick) were dewaxed in xylene and rehydrated through graded concentrations of ethanol to distilled water. Sections were then immersed in 10 mmol/L citrate buffer (pH 6.0) and processed in thermostatic water bath for 30 min at 98 °C for antigen retrieval. For staining with CD31-specific antibody, the tumor tissue sections were blocked overnight in 5%BSA/PBS at 4 °C. Afterword, the sections were incubated with anti-CD31 antibody (1:100) for 4 h at room temperature. For PSMA-specific antibody, consecutive tumor tissue sections were blocked for 2 h at room temperature in 10% goat serum/PBS/0.2% TX-100. Subsequently, the sections were exposed overnight at 4 °C to the PSMA-specific antibody (1:250). Finally, the sections were incubated for 1 h at room temperature with DyLight488-labeled antibody and coated with DAPI-containing media VectaShield. To distinguish blood vessels from other tissue components, corresponding tumor tissue sections were stained with Masson’s Trichrome stain according to supplied protocol (Sigma-Aldrich).

### Statistical analysis

Cellular uptake experiments and all flow cytometry analyses were performed twice in triplicate and by repeating independent blocks of experiments including all appropriate controls. Data are presented as mean ± standard deviation. The percentage of specific cell death was calculated as 100% × (experimental dead cells [%] − spontaneous dead cells in medium [%])/(100% − spontaneous dead cells in medium [%]). All statistical calculations were performed using GraphPad Prism v.6.00 (La Jolla, CA, USA) for Windows. Data were analyzed using 1- and 2-way ANOVA with post hoc comparisons performed using the ˇSid´ak correction, Dunnett’s test, and Tukey’s tests. Values of *P* < 0.01 were considered to be statistically significant.

## Results

### Triple-negative breast cancer cells promote vessel formation and induces PSMA expression on endothelial cells

**Fig. 1:**
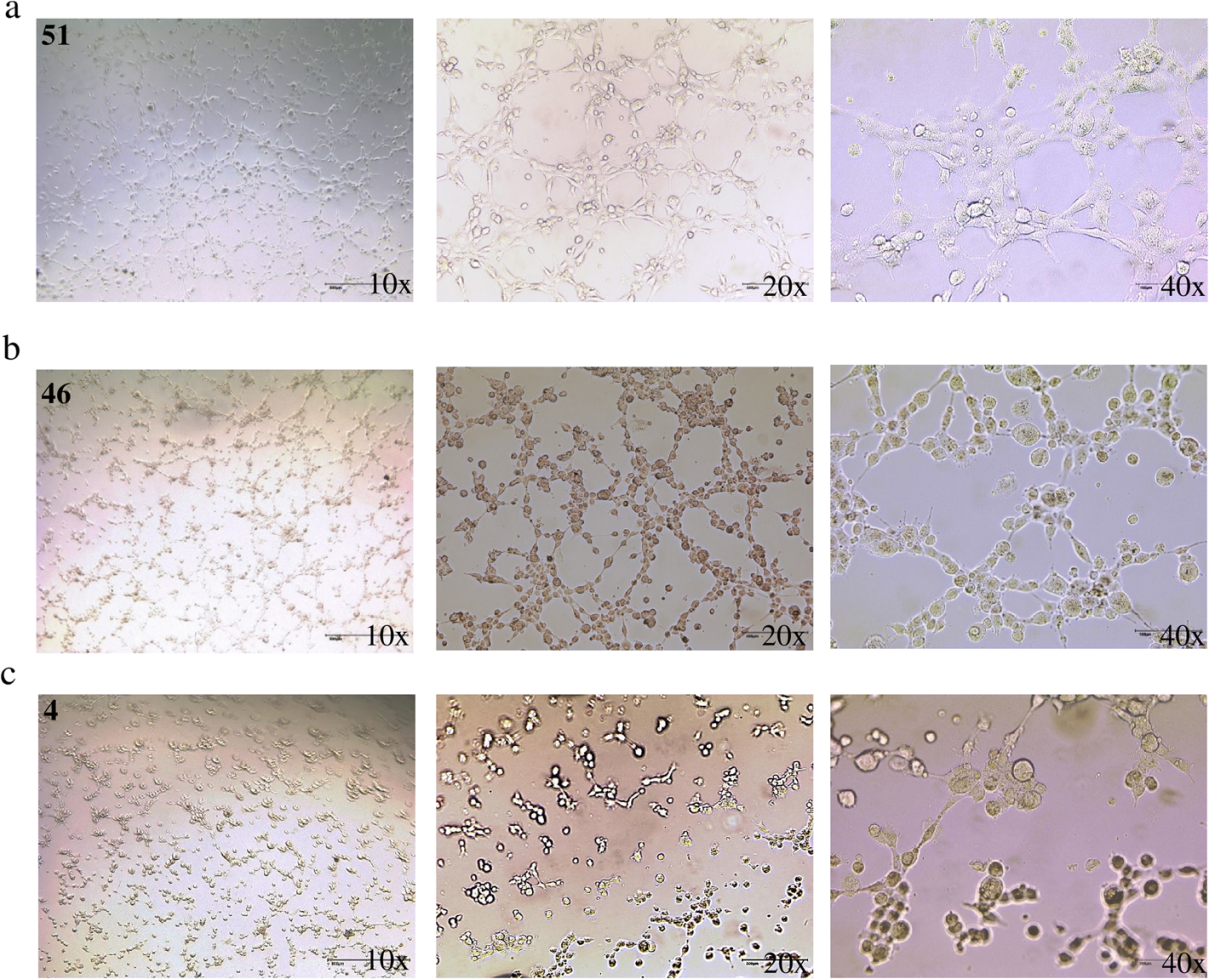
TNBC cells promote tube formation by endothelial cells. Bright field microscopy imaging of tube formation efficiency of HUVEC cells incubated in **a** EBM-2 growth medium supplemented with endothelial growth factors, **b** MDA-MB231-conditioned medium, and **c** MCF-7-conditioned medium

**Fig. 2:**
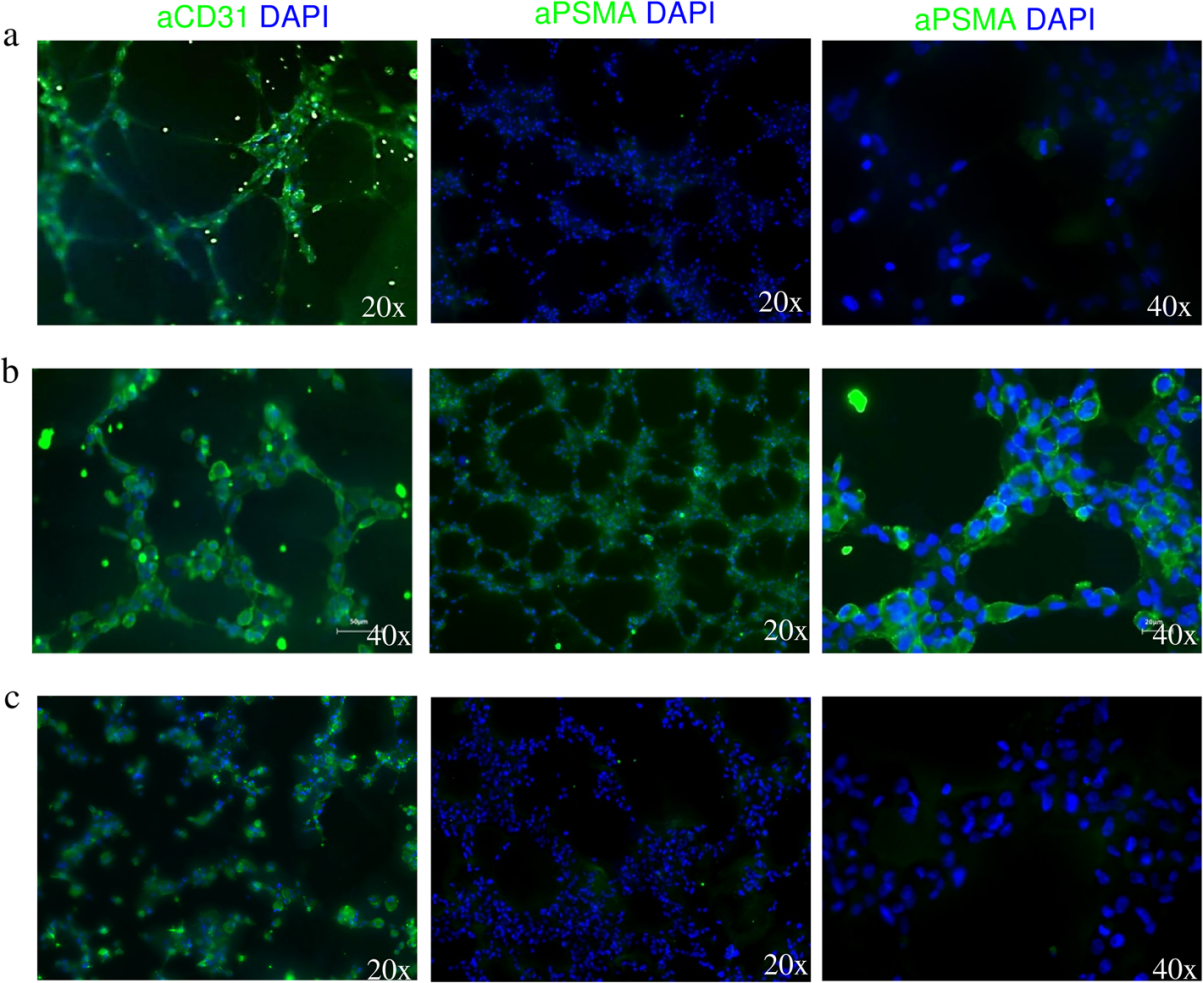
TNBC cells induce PSMA expression on endothelial tubes. Fluorescence microscopy analysis of tube-associated expression of CD31 and PSMA on HUVEC cells incubated in **a** EBM-2 growth media supplemented with endothelial growth factors, **b** MDA-MB231-conditioned medium, and **c** MCF-7-conditioned medium

The triple-negative breast cancer cell line MDA-MB231 induced HUVEC cell to generate tubes as efficiently as the growth factors VEFG, EGF, and FGF containing EBM-2 medium (46 vs. 51 tubes for MDA-MB231-conditioned medium and EBM-2 medium, respectively, Fig. 1a, b). In contrast, virtually, no tube formation was detected for HUVECs incubated in the MCF-7-conditioned medium (non TNBC cells, Her2^−^, PR^−^, ER^+^) (Fig. 1c). As indicated by fluorescence microscopy, all endothelial cells expressed the specific CD31 marker, regardless of medium source (Fig. 2a–c). However, solely, the TNBC-conditioned medium induced PSMA expression in the endothelial cells.

### PSMA presents an addressable target on breast cancer cells and TNBC-associated endothelial cells

**Fig. 3:**
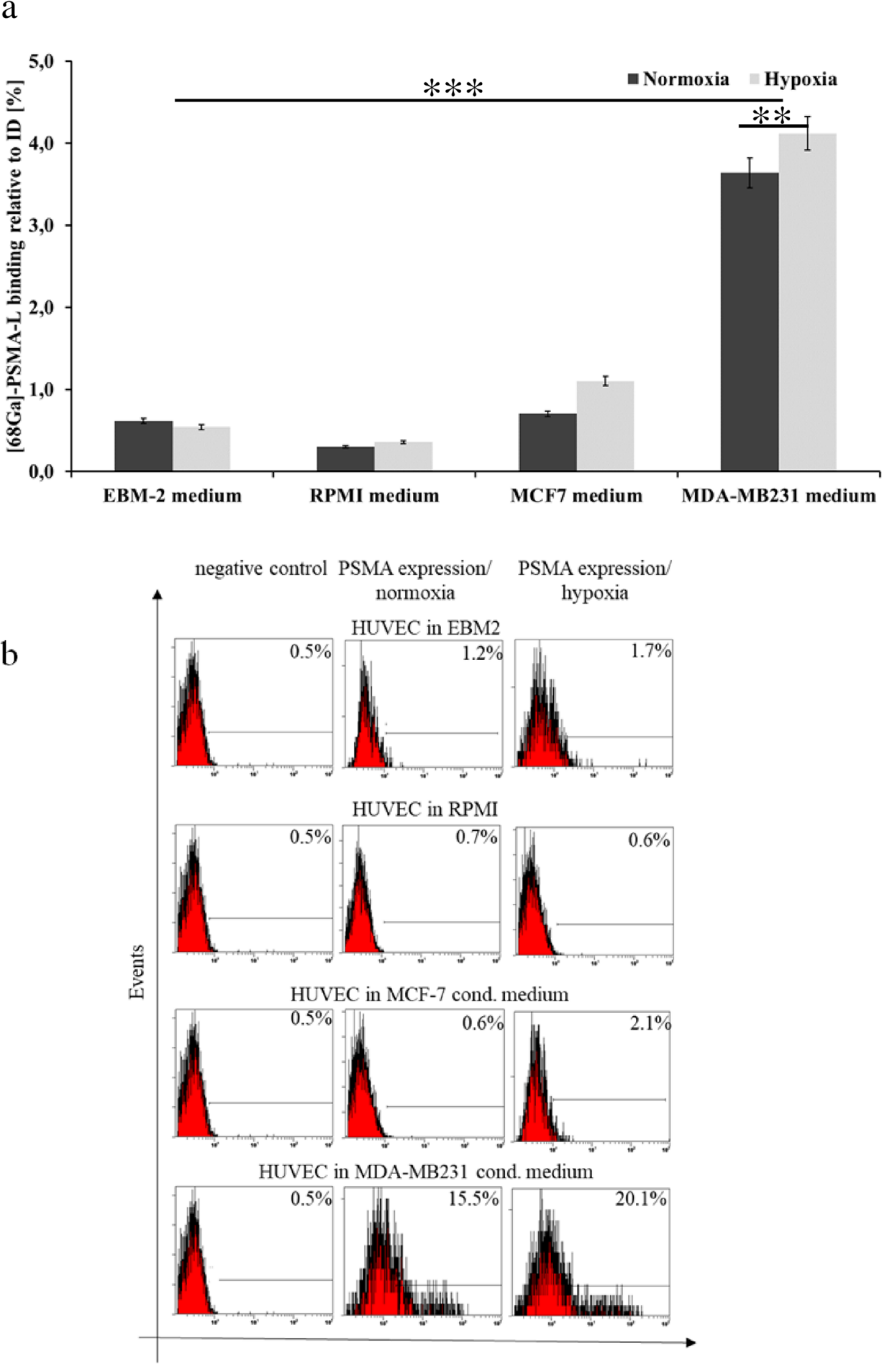
[^68^Ga]-PSMA-11 binding correlates with target expression on endothelial HUVEC cells. **a** Cellular binding (1 h) of [^68^Ga]-Ga-PSMA-11 on HUVEC cells incubated in different medium under normoxic and hypoxic conditions. **b** Corresponding flow cytometric analysis of PSMA expression in HUVEC cells. Data are representative of two independent experiments with triplicates (*n* = 6). Bars, ± SEM. **p < 0.01, ***p < 0.001

**Fig. 4:**
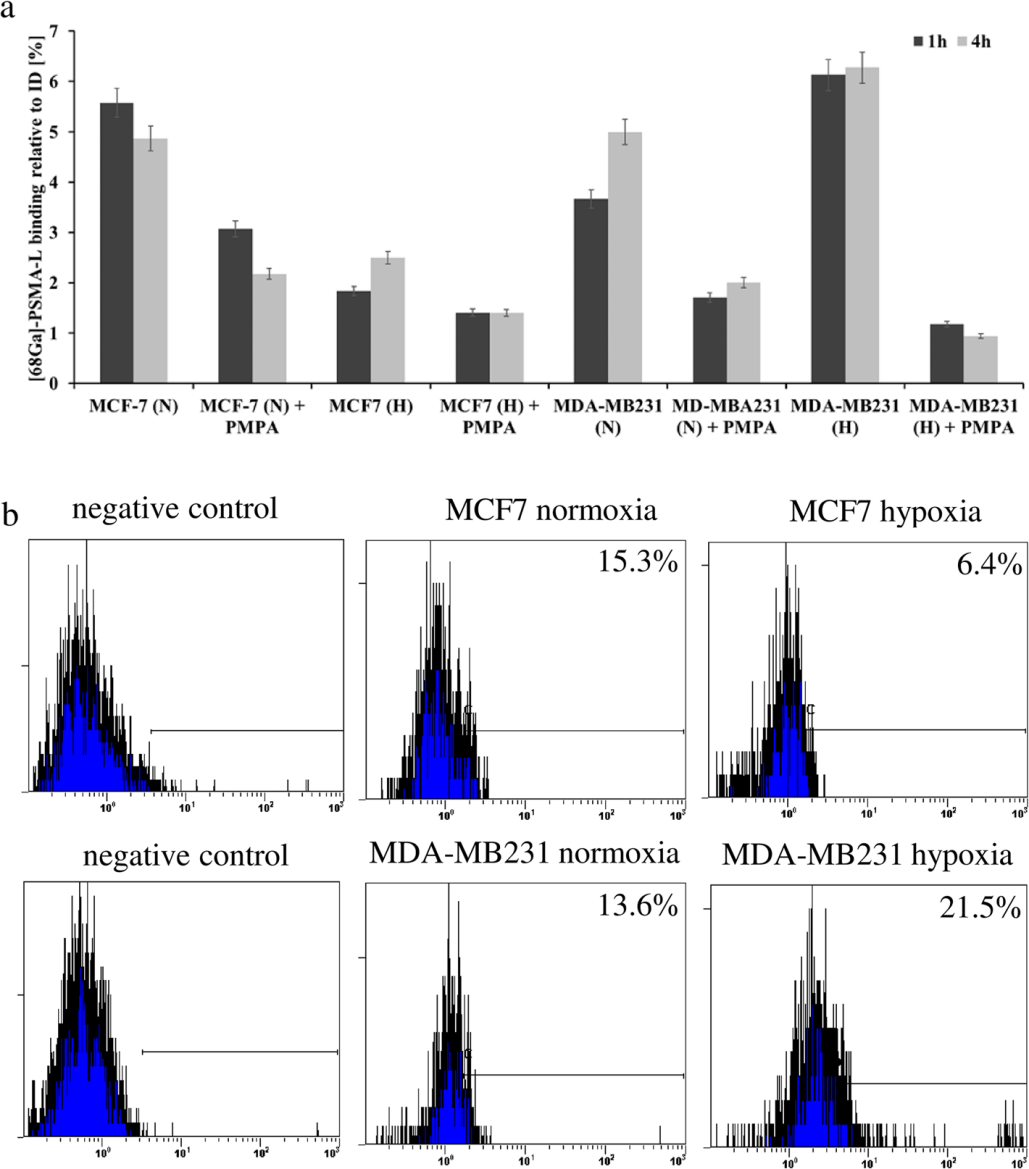
[^68^Ga]-PSMA-11 binding correlates with target expression on breast cancer cells. **a** Cellular binding of [^68^Ga]-Ga-PSMA-11 on breast cancer cells under normoxic (N) and hypoxic (H) conditions with and without PAMP inhibitor. **b** Corresponding flow cytometric analysis of PSMA expression. Data are representative of two independent experiments with triplicates (*n* = 6). Bars, ± SEM

As indicated by a gamma counter analysis, HUVEC cells cultured in MDA-MB231 cell medium showed significantly higher binding of ^68^Ga-labeled PSMA-L than MCF-7-condined, EBM-2, or control RPMI growth media (3.64% vs. 0.70%, 0.62%, 0.30%, respectively, *p* < 0.0001; Fig. 3a). Interestingly, the binding of [^68^Ga]-Ga-PSMA-11 on TNBC-conditioned endothelial cells further increased under hypoxic conditions. These binding data correlated with PSMA expression as indicated and quantified using a flow cytometric analysis (Fig. 3b). Additional to HUVEC cells, the breast cancer were evaluated directly for their addressability with PSMA-specific tracer. The [^68^Ga]-Ga-PSMA-11 bound efficiently on TNBC cells MDA-MB231 as well on the NTNBC cells MCF-7 (Fig. 4a). However, contrary to time-dependent tracer binding increase in MDA-MB231 cells, for MCF-7 cells, a declined radioactivity accumulation was detected after 4 h of incubation (3.66% vs. 5.00% and 5.58% vs. 4.87% for MDA-MB231 and MCF-7 cells after 1 h and 4 h, respectively, *p* < 0.01). As detected for HUVEC cells, incubation with [^68^Ga]-PSMA-11 under hypoxic conditions significantly increased tracer binding on the TNBC cells (3.66% and 5.00% vs. 6.13% and 6.27%, for normoxic and hypoxic conditions, after 1 h and 4 h, respectively, *p* < 0.001). Contrary, the binding of [^68^Ga]-PSMA-11 on MCF-7 cells dropped from 5.58% and 4.87% to 1.83% and 2.50%, after 1 h and 4 h, respectively (*p* < 0.001). The co-incubation with PSMA-specific inhibitor PMPA significantly lowered the tracer binding on PSMA. As verified by the flow cytometry, the tracer binding clearly correlated with the PSMA expression under normoxic and hypoxic conditions on both cell lines (Fig. 4b).

### [^177^Lu]-PSMA-617 acts anti-angiogenically on TNBC-associated endothelial tubes

**Fig. 5:**
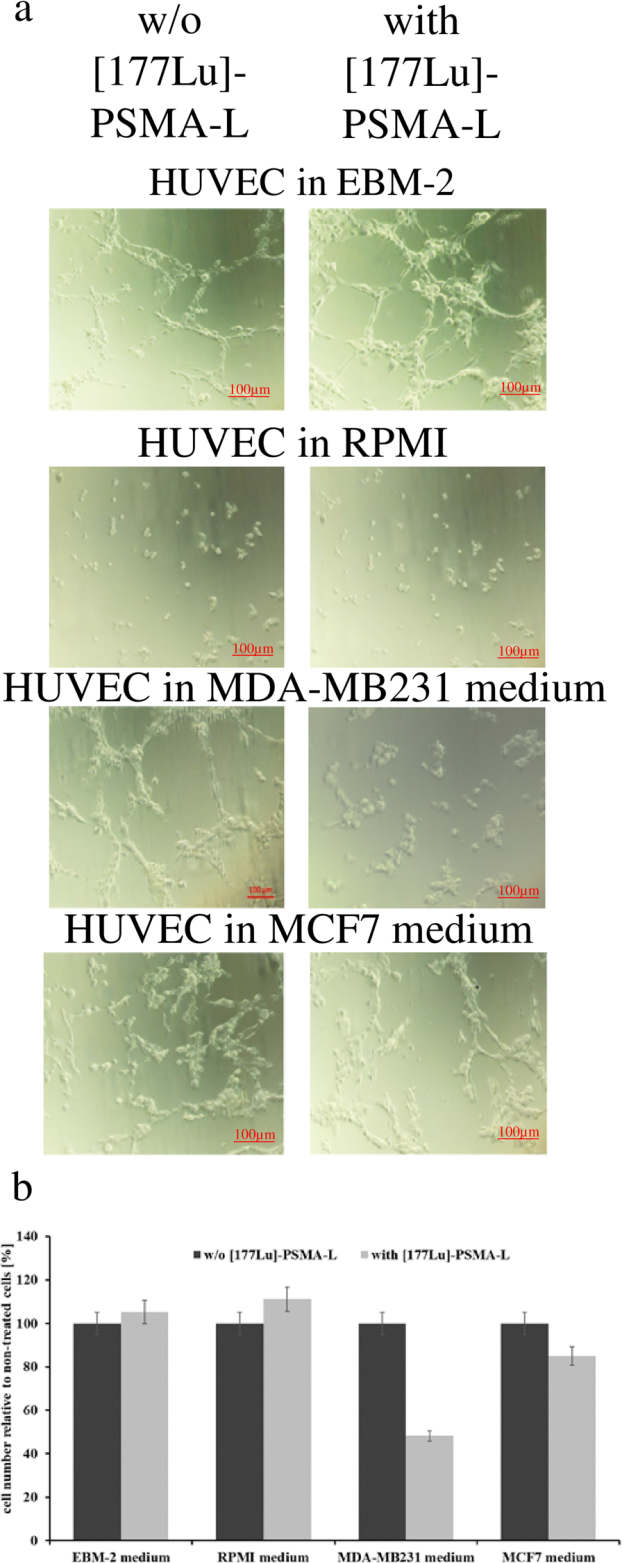
[^177^Lu]-PSMA-617 decreases viability of TNBC-conditioned HUVEC cells and impairs their potential for tube formation. **a** Bright field microscopy imaging of tube formation efficiency of HUVEC cells incubated without or with [^177^Lu]-PSMA-617 in different medium. **b** HUVEC cell vitality after incubation in different media without or with [^177^Lu]-PSMA-617. Data are representative of two independent experiments with triplicates (*n* = 6). Bars, ± SEM

As visualized by the bright field microscopy, ^177^Lu-labeled PSMA-L efficiently targeted the HUVEC cells. The most pronounced destroying effect was detected in TNBC-conditioned cells with massive destruction of tubes’ architecture (Fig. 5a). For EBM-2-conditioned cells, the vessels’ integrity remained unaffected. Corresponding to the bright field imaging, the highest rate of apoptosis induced by [^177^Lu]-PSMA-617 was detected in HUVEC cells conditioned with the MDA-MB231 medium (48.15% viable cells, Fig. 5b). For the MCF-7-conditioned HUVEC cells, the viability decreased by 15%.

### [^68^Ga]-PSMA-11 efficiently accumulates in TNBC tissue in vivo

**Fig. 6:**
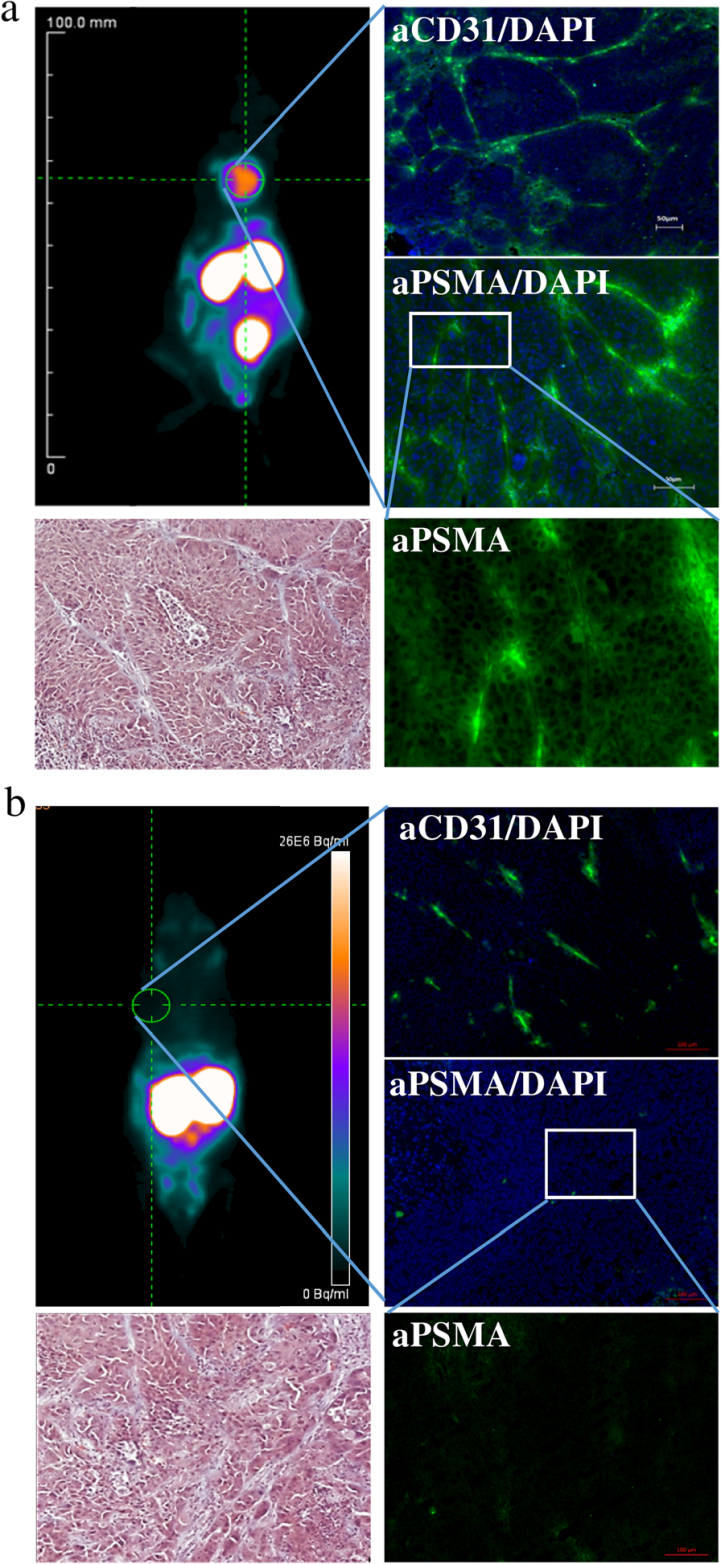
PSMA-addressing tracer accumulates efficiently and specifically in PSMA-expressing tumor tissue. **a** μPET analysis of [^68^Ga]-PSMA-11 biodistribution 30 min p.i. in MDA-MB231 subcutaneously xenografted NUDE mice with fluorescence microscopy imaging of tumor tissue section stained with CD31- and PSMA-specific antibodies and DAPI, and with Masson’s Trichrome stain. **b** μPET analysis of [^68^Ga]-PSMA-11 biodistribution 30 min p.i. in MCF-7 subcutaneously xenografted NUDE mice with fluorescence microscopy imaging of tumor tissue section stained with CD31- and PSMA-specific antibodies and DAPI, and with Masson’s Trichrome stain (*n* = 5)

The μPET acquisition performed 30 min post intravenous injection in mice subcutaneously transplanted with MDA-MB231 or MCF-7 cells indicated the target selectivity and the targeting potential of [^68^Ga]-PSMA-11 (Fig. 6). The high target specificity demonstrated in this and other study [11] resulted in highly specific tracer accumulation in MDA-MB231 tumor with a T/B ratio of 43.3 ± 0.9. Staining of tumor tissue section with CD31- and PSMA-specific antibodies visualized the tumor-associated blood vessels and the PSMA expression on endothelial as well as on cancer cells (Fig. 6). Corresponding to the μPET image, no PSMA was detected neither on endothelial nor on the MCF-7 cells (T/B ratio of 1.1 ± 0.1). The tracer accumulation detected in the kidneys is due to its renal extraction and PSMA expression in the kidney, shown to be highly increased in rodents [12].

## Discussion

In adults, angiogenic processes are linked to pathological states, like tumor growth and metastasis [13]. Consequently, several anti-vascular therapeutic approaches using antibodies and small molecule inhibitors have been developed and clinically evaluated [14]. Though some of them have yielded promising outcomes, the overall efficiency needs to be improved to avoid cytotoxic off-site effects and to overcome the inherent and acquired resistance mechanisms [15]. Importantly, in most cases, a positive effect could be observed only when the anti-angiogenic drugs were given in combination with cytotoxic drugs or targeted molecules [16, 17]. Thus, linking angiogenesis-addressing molecules with an on-site endogenous irradiation presents an attractive therapeutic option provided existing tumor vasculature expresses direct targetable structure. PSMA was primarily described as a marker highly overexpressed on epithelial prostate cancer cells [18]. Meanwhile, PSMA-addressing urea-based derivatives are clinically used for molecular imaging and endogenous radio ligand therapy of PrCa [19–22]. PSMA has been detected on non-prostatic malignancies; however, the expression of PSMA has been described to be restricted to the endothelial cells of the neovasculature with no expression on adjacent normal endothelium. Thus, neovasculature-associated PSMA and its addressing provide a promising therapeutic approach especially of the so-called non-targetable cancer entities. Among them, the triple-negative phenotype of breast cancer is deemed to be the most aggressive and lethal malignancy in female population worldwide [23]. Several case reports or series have shown the potential of PSMA-targeted imaging in breast cancer among which also cases of TNBC [24–26]. A detailed evaluation of the expression on the tumor cells itself and the endothelium of the tumor vessels revealed a higher expression on the endothelium of the tumor vessels, with higher expression rates especially in TNBC in comparison to other breast cancer entities [25, 27]. In this study, we evaluate systematically and show for the first time in vitro and in vivo the potential of targeting of neo-vasculature-associated PSMA by a radiolabeled PSMA-ligand as a promising therapy option in TNBC.

PSMA is a glutamate carboxypeptidase II with folate hydrolase 1 (FOLH1) and *N*-acetylated α-linked acidic dipeptidase (NAALADase) activities [28, 29]. The carboxypeptidase activity and an expression pattern analogue to that of pro-angiogenic acting CD13/APN molecule strongly suggest the regulating role of PSMA during the angiogenic processes in tumor tissue [30, 31]. As shown by Conway et al., PSMA participates in laminin-specific integrin signaling and in regulation of cytoskeletal dynamics which are required for angiogenesis in vivo and for invasiveness of endothelial cells in vitro. Recent in vitro studies suggest the endothelial PSMA expression to be induced by tumor-released factors [32, 33]. In this study, we found that the TNBC cells MDA-MB231 led the endothelial cells to generate tubes at a level comparable to VEGF-containing medium. In contrast, the NTNBC cells MCF-7 did not show any pro-angiogenic effect, suggesting that the stimulating potential is not related to the PSMA expression. These data correspond to the pathological profile of TNBC characterized by aggressive and highly metastatic progression. As shown by Wang et al., the TNBC cells produce higher level of VEGF and MMP-9 which are known to activate the vascular endothelial cells and initiate the angiogenesis process [34, 35]. Moreover, PSMA was shown to work downstream of MMP-2 and MMP-14 generating laminin peptide fragments that activate endothelial cell adhesion and induce angiogenesis in vivo [36]. Interestingly, as detected by fluorescence microscopy and flow cytometric analysis solely the TNBC cells induced PSMA expression on the generated tubes. Previous in vivo studies suggested PSMA to be crucial for endothelial function in pathologic angiogenesis [37]. By regulation of signal cascade involving p21-activated kinase 1 (PAK-1) and focal adhesion kinase (FAK), PSMA impairs fundamental angiogenic steps like adhesion, motility, and invasion of endothelial cells. Consistent with the proangiogenic function of PSMA, the PSMA-negative tumors were smaller, of lower-grade, and more apoptotic with fewer blood vessels [38]. Interestingly, under hypoxic conditions, PSMA expression increased significantly on the TNBC-conditioned HUVEC cells as well as directly on MDA-MB231 cells, providing by this a higher number of cellular targets for radiolabeled PSMA-L. In support of these findings, as shown in PSMA wild-type and PSMA knock out TRAMP mouse models, PSMA expression mediates hypoxia tolerance in tumor cells [38]. Surprisingly, the vessels lacking PSMA were more normalized and regular, while vessels in wild-type tumors were irregularly branched and dilated, an architecture typical for tumor-associated neo-vasculature. However, the PSMA-negative tumors were smaller, of lower-grade, and with fewer blood vessels consistent with the proangiogenic function of PSMA and suggesting that endothelial PSMA contributes to the augmented and dysregulated vessel growth [38]. Although the prostate cancer in PSMA^−/−^ mice were less hypoxic than their wild-type counterparts, the viable cell areas in PSMA^+/+^ tumor were more than 30% larger. Apparently, PSMA-expressing tumor cells remained viable at oxygen concentrations that are toxic for normal cells and can survive distant to the tumor vasculature despite increased tissue hypoxia. PSMA expression in tumor cells appears to confer an intrinsic survival advantage that is more beneficial to tumor growth than a favorable vessel phenotype. As we previously have shown, TNBC cells response to hypoxic condition by upregulation of hypoxia-inducible factor-1α (HIF-1α) expression [39]. HIF-1α plays the most important part in activation of vascular endothelial cells and initiation of angiogenesis processes under hypoxia by induction of expression and release of VEGF [40]. This represents one of the mechanisms behind the high therapy resistance of the TNBC.

Our therapeutic study with [^177^Lu]-PSMA-617 visualizes the potential of PSMA as a unique and attractive target for the TNBC. As detected by vessel formation assay, incubation with [^177^Lu]-PSMA-617 was highly apoptotic for MDA-MB231-conditioned HUVEC cells leading to a massive tube destruction. The mean penetration range of β^−^ particles emitted by ^177^Lu in soft tissue is 670 μm. Moreover, this radionuclide induces bystander and cross-fire effects [41]. Owing to both effects, there is no need to address each single tumor cell, thereby allowing induction of an apoptotic effect in tumors with reduced perfusion and with heterogeneous target distribution [42]. Molecular imaging using μPET confirmed the targetability of TNBC with PSMA-addressing ligands in vivo. Consistently with in vitro data, [^68^Ga]-PSMA-11 efficiently accumulated in PSMA-expressing MDA-MB231 xenograft. The homogenous intratumoral tracer distribution results from the endothelial and epithelial expression of PSMA, as confirmed by a subsequent tissue staining analysis. This expression pattern allows targeting of two tumor tissue compartments at the same time, the neo-vasculature and TNBC epithelial cells. In contrast to general angiogenetic targets like VEGF or integrin, endothelial PSMA expression is specific for tumor-associated neo-vasculature. This and its epithelial expression make PSMA an ideal target for endogenous radiotherapy of TNBC. Moreover, as shown for PSMA-expressing endothelial and malignant cells, binding on PSMA induces an efficient ligand internalization [32, 43]. From therapeutic point of view, this will allow intracellular drug delivery and trapping, which would increase intracellular dose accumulation. The coexisting expression of PSMA detected in the TNBC tumor might be linked to its enzymatic activities which promote and contribute to different pathological processes. As previously shown for the endothelial compartment, PSMA is acting as *N*-acetylated α-linked acidic dipeptidase, which by cleavage of signaling molecules is involved in angiogenesis [5, 6, 32, 36]. Additionally, based on our previous study, PSMA may also facilitate neo-vasculature processes as folate hydrolase by increasing the local availability of folic acid, which is crucial for the adequate function of the endothelial nitric oxide synthase [44, 45]. For TNBC cells, PSMA is essential for the cancer cell mobility and invasiveness, as described for the prostate cancer cells [46, 47]. However, the hypoxia-increased expression of PSMA on the TNBC cells suggests PSMA playing a major role in adaptation to environmental conditions. As we previously have shown, TNBC is particularly capable of increasing intracellular concentration of glutathione (GSH), the most abundant antioxidant protecting against ROS and regulating the intracellular redox status [39]. GSH is a tripeptide consisting of glycine, cysteine, and glutamate. Due to the *N*-acetylated α-linked acidic dipeptidase activity, PSMA generates glutamate, which is efficiently taken up by the glutamate auxotroph TNBC cells [48]. Moreover, TNBC cells have been shown to be cysteine addicted which explains the increased expression of the cysteine transporter CD44iv/xCT on their cell surface [49]. Considering this, PSMA may contribute to intracellular generation of GSH which regulate oxidative stress and by this increase the therapy resistance of TNBC.

## Conclusion

In this study, we present the rationale of endogenous radio-ligand therapy targeting PSMA for treatment of TNBC. The coexisting expression on the endothelial as well as on the epithelial cells of TNBC provides an ideal therapeutic target allowing simultaneous addressing of two tumor compartments.

## Data Availability

All data generated or analyzed during this study are included in this published article.

## Abbreviations

BRCA: Breast cancer gen
ERT: Endogenous radiotherapy
FOLH-1: Folate hydrolase 1
GSH: Glutathione
HIF-1: Hypoxia-induced factor 1
MMP: Matrix metalloproteases
NAALDase: *N*-Acetylated α-linked acidic dipeptidase
PARP: Poly(ADP-ribose) polymerase
PET/CT: Positron emission tomography/computer tomography
PrCa: Prostate cancer
PSMA: Prostate-specific membrane antigen
RLT: Radio-ligand therapy
TNBC: Triple-negative breast cancer
T/B ratio: Tumor to background ratio
VEGF: Vascular endothelial growth factor
^177^Lu: 177-Luthecium
^68^Ga: 68-Gallium
